# Optic radiation atrophy in Lewy body disease with visual hallucination on phase difference enhanced magnetic resonance images

**DOI:** 10.1038/s41598-022-21847-4

**Published:** 2022-11-03

**Authors:** Mari Miyata, Shingo Kakeda, Tetsuya Yoneda, Satoru Ide, Kazumasa Okada, Hiroaki Adachi, Yukunori Korogi

**Affiliations:** 1 Department of Radiology, University of Occupational and Environmental Health, School of Medicine, 1-1 Iseigaoka, Yahatanishi-Ku, Kitakyushu, Fukuoka 807-8555 Japan; 2 Department of Radiology, Hirosaki University Graduate School of Medicine, Aomori, Japan; 3 Department of Medical Physics in Advanced Biomedical Sciences, Faculty of Life Sciences, Kumamoto University, Kumamoto, Japan; 4 Department of Neurology, School of Medicine, University of Occupational and Environmental Health, Kitakyushu, Japan

**Keywords:** Neuroscience, Diseases, Neurology

## Abstract

Visual hallucinations (VH) occur commonly in Lewy body disease (LBD), including Parkinson’s disease (PD), PD with dementia, and dementia with Lewy bodies. We aimed to use phase difference enhanced imaging (PADRE) to assess structural abnormalities of optic radiation (OR) in patients with Lewy body disease (LBD) concomitant with VH. Firstly, two radiologists reviewed the OR appearances in healthy subjects (HS) on PADRE. Next, based on the OR abnormalities, two reviewers assessed the PADRE images from 18 HS and 38 and 110 patients with LBD, with and without VH, respectively, in a blinded manner. Finally, all patients with LBD without VH were eventually followed up for at least 5 years after magnetic resonance imaging to determine the appearance of VH. The radiologists identified three layers, namely external sagittal stratum, internal sagittal stratum, and tapetum, in OR on the PADRE in HS. Moreover, they were able to consensually define the OR as abnormal when the layers were obscured and the disappearance of the cranial side. The sensitivity/specificity of abnormal OR for each case was 68%/81% (LBD with VH vs. LBD without VH). Furthermore, VH appeared in 12 of the 21 (57%) patients with LBD and abnormal OR during the follow-up period. However, no patients without abnormal OR reported VH. Patients with LBD and VH demonstrated the abnormal OR. This, in turn, might be a useful marker to distinguish the patients with VH from those without VH and HS. Moreover, abnormal OR on PADRE may precede the appearance of VH in LBD.

## Introduction

Dementia with Lewy bodies (DLB) is the second most common neurodegenerative dementia in the elderly after Alzheimer’s disease (AD)^1^. Considering the lack of major clinical differences between Parkinson’s disease (PD) with dementia (PDD) and DLB^2–4^, both are classified as different forms of Lewy body disease (LBD)^2^. The majority of patients with DLB have mixed AD and Lewy body pathology^2^. Hence, this distinction is clinically relevant because it is often difficult to clinically distinguish patients with mixed pathology from those with AD^2^. Visual hallucinations (VH) are common in PDD or DLB. Moreover, the presence of VH is predictive of debilitating conditions, such as those related to cognitive impairment, institutionalization, and higher mortality, compared to patients without VH^5^. Despite no difference in the rate of cognitive decline, DLB increases the risk of mortality, compared to AD^6^. The precise nosological differences between DLB and AD remain uncertain. Nonetheless, VH tend to emerge early in the course of the disease. Hence, they a useful diagnostic feature in DLB^2^. Moreover, the shorter clinical course in DLB underscores the importance of an accurate antemortem diagnosis. Thus, it is important to predict the occurrence of VH.

The physiopathology underlying VH in patients with LBD is not well understood. Decreased visual acuity has been associated with VH in patients with PD^7^. Furthermore, such patients with VH experience retinal nerve fiber layer (RNFL) thinning^8^. A previous neuroimaging study reported an increase in diffusivity in optic radiation (OR) in patients with PD and VH, compared to that in those without VH and healthy controls^9^. Therefore, structural abnormalities in visual association areas, including OR might be associated with VH in patients with LBD.

Phase difference enhanced (PADRE) imaging is the new phase-weighted magnetic resonance imaging (MRI) technique. It selects the phase difference between the target and surrounding tissue to enhance the contrast of the target tissue^10,11^. Some PADRE images offered a new level of contrast that could not be realized with earlier phase techniques^10,11^. Moreover, various fiber tracts, such as the OR and central tegmental tract, were delineated on the high spatial resolution PADRE images^10,11^. Therefore, we hypothesized that PADRE might facilitate the visualization of pathological changes of OR in patients with LBD and VH. We intended to assess the signal intensity of the OR in patients with LBD and VH on PADRE, compared to healthy subjects (HS) and patients with LBD without VH. Moreover, we aimed to examine the relationship between abnormal findings in the OR on PADRE and VH in patients with LBD.

## Methods

### Approval

This study protocol was approved by the Institutional Review Board at the University of Occupational and Environmental Health School of Medicine (Kitakyushu, Fukuoka, Japan, UOEHCRB22-013) and was conducted in accordance with the Declaration of Helsinki. Imaging and clinical data were retrospectively acquired from 148 patients with Lewy body disease and 18 healthy subjects. The Ethics Committee of the University of Occupational and Environmental Health granted a permission to use the retrospective data in the study without individual informed consent.

### Image acquisition

All studies were performed on a 3 Tesla MRI system (Signa EXCITE 3T; GE Healthcare) using a dedicated eight-channel phased-array coil (USA Instruments Aurora, OH, USA). All patients and HS underwent brain MRI according to our standard protocol, including T2-weighted images (T2WI) and fluid attenuated inversion recovery (FLAIR) imaging. T2WI and FLAIR imaging were obtained on the axial planes. T2WI parameters included the following: repetition time (TE), 4500 ms; repetition time (TR), 58.4 ms; number of excitations (NEX), 1; and imaging time, 2 min 10 s. FLAIR parameters included the following: TE,12,000 ms; TR, 140 ms; inversion time, 2600 ms; NEX 2; and imaging time, 2 min 10 s. T2WI and FLAIR images were acquired at a section thickness of 4 mm, an intersection gap of 2.5 mm, a field of view of 22 cm and a matrix of 256 × 192. A 3-D flow-compensated multi-echo spoiled gradient echo (GRE) sequence was used to obtain PADRE images. The imaging parameters included the following: coronal planes covering the brain; number of echo times (TE), 11; first TE, 4.5 ms; uniform TE spacing, 5 ms; repetition time, 58.4 ms; flip angle, 15°; bandwidth per pixel, ± 62.5 Hz; field of view, 22 × 16.5 cm; acquisition matrices, 320 × 416; slice thickness, 1.5 mm; and imaging time, 7 min 1 s.

### PADRE technique

The description of the PADRE imaging technique can be found in a previously published study^10^. The “phase difference selection” is one of the major concepts responsible for the power of the PADRE technique. It enhances the magnetic properties of the target tissue. PADRE classifies and selects various phase differences, $\Delta \theta$, to enhance different tissues. Moreover, it enhances all of them on the magnitude image $\left| \rho \right|$ by the enhancing function $w(\Delta \theta )$. The PADRE image $\rho_{PADRE}$ was reconstructed using the following formula:

$$ \rho_{PADRE} = w(\Delta \theta )\left| \rho \right|. $$

In this study, we selected the positive phase, $\Delta \theta$, in the right-handed system to primarily enhance the brain structure related to myelin rich tissues. The reconstitution parameters of PADRE were optimized according to previously published results^10^.

### Subjects

This retrospective study design was approved by the institutional review board. Thus, the requirement for informed consent was waived off. The 3-D multi-echo spoiled GRE sequence has been a part of routine brain MRI in our institution since May 2012. It has been primarily used for the following indications: (1) screening of a minor hemorrhage and (2) the evaluation of vascular disease, movement disorder, or degenerative disease. The exclusionary criteria were as follows: (i) atypical or secondary forms of parkinsonism (e.g., multiple system atrophy, progressive supranuclear palsy, corticobasal degeneration, or parkinsonism because of neuroleptic exposure, cerebrovascular disease, or known structural causes); (ii) other medical or neurological reasons for cognitive impairment (e.g., seizures, strokes, head trauma, significant vision, or hearing deficits); or (iii) contraindications to MRI (e.g., surgical clips or foreign metallic implants). All patients with PDD had motor symptoms for at least 1 year before the onset of dementia. First, two radiologists (with 23 and 8 years of experience in neuroradiology) selected 93, 20, 35 patients with PD, PDD, DLB, and 18 HS, respectively, from the MRI database in Dec 2018. All patients were diagnosed by one of the authors with an experience of 30 years in movement disorders. All patients with PD fulfilled the Movement Disorder Society criteria^12^ for the diagnosis of idiopathic PD. Those with DLB were diagnosed with probable DLB, according to the Consortium on DLB International Workshop in 2017^13^. Furthermore, patients with PDD fulfilled the diagnostic criteria for possible PDD, published by the Movement Disorder Society in 2007^3^. The medical records of all subjects were reviewed to capture the description of complex VH (involuntary images about people, animals, and objects that are experienced as real during the waking state but for which there is no objective reality^14^), including all tenses of ‘see’ appeared. Thus, in thirty-eight patients with LBD, the presence of VH was identified before undergoing MR imaging. We separated the set of patient data into the following two groups: LBD with VH (9, 28, and one patient with PDD, DLB, and PD, respectively) and LBD without VH (11, seven, and 92 patients with PDD, DLB, and PD, respectively). Furthermore, for patients with LBD without VH before MRI, we identified in the medical record whether there was any mention of the appearance of complex VH during the 5-year follow-up period from the date of MRI.

Moreover, we selected 18 age- and sex-matched HS (11 women, 7 men; mean age 68.0 years, range 51–86 years) without a history of neurological or psychiatric diseases from the aforementioned database. This facilitated an evaluation of the normal appearance of the OR. Indications for their examination included headache, anterior communicating and middle cerebral artery aneurysms, bilateral upper extremity numbness, and benign positional vertigo. The conventional MR imaging results were normal in all subjects.

We reviewed the disease duration of LBD (interval between the initial onset of LBD and the brain MR study) and medication (the presence or absence of Parkinson’s disease and/or dementia drugs). Furthermore, cognitive impairment was assessed by the revised version of Hasegawa’s Dementia Scale (HDS-R) and/or the Mini-Mental State Examination (MMSE) in LBD patients. The test scores were below the cutoff points of 20/21 for the HDS-R^15^, and 23/24 for the MMSE^16^ were considered positive for cognitive impairments. If both MMSE and HDS-R were performed, a patient was considered positive for cognitive impairment if either was below the cutoff value. These cognitive assessments were performed on the patients with presented or suspected dementia.

## Image interpretation

### Nonblinded PADRE evaluations

The neuroradiologists evaluated the normal appearance of the OR on PADRE images. They evaluated the findings of the OR in HS. Following the evaluation of the normal appearances of the OR, they recorded any deviations from the normal anatomy as abnormal findings observed in five patients with LBD and VH (three and two patients with DLB and PDD, respectively).

### Blinded PADRE evaluation

**Figure 1:**
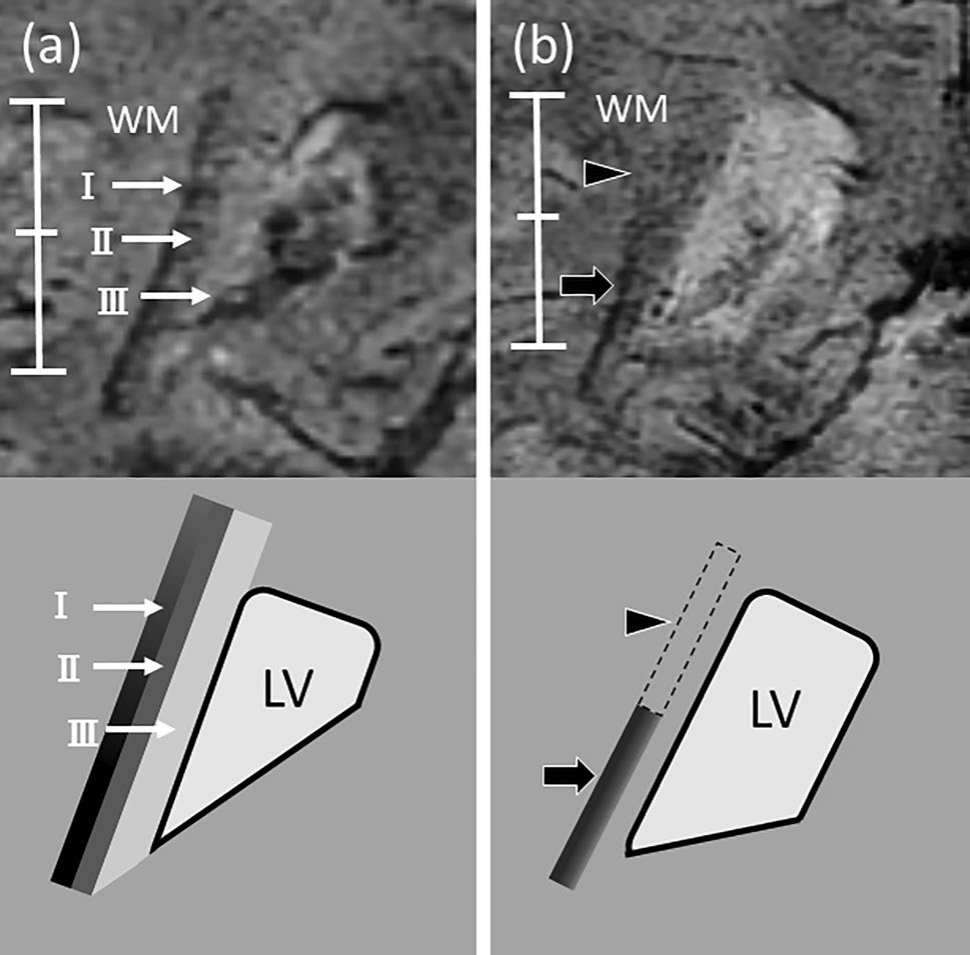
Coronal phase difference-enhanced imaging images from (**a**) healthy subjects and (**b**) patients with dementia with Lewy bodies and visual hallucinations. (**a**) The delineation of three layers at the right optic radiation (OR) (I: external sagittal stratum, II: internal sagittal stratum, and III: tapetum) in a healthy subject (a 62-year-old woman). (**b**) The obscuration of three layers in the OR (black arrowhead) and the disappearance of the cranial side of the OR (black arrow) in a patient with Dementia with Lewy bodies and visual hallucinations (a 69-year-old woman).

First, two reviewers (both with 12 years of experience in neuroradiology) independently reviewed the PADRE images. Ten training cases (5 each for patients with LBD and VH and 5 HS) were presented before the tests. Moreover, they underwent sufficient training on the PADRE images to gain familiarity with the following normal and abnormal appearances: (a) an obscuration of the inner layer and outer layer in the OR and (b) the disappearance of the cranial side of the OR (Fig. 1, see results). The following four groups were defined based on the patterns of abnormal findings in the OR: group 1, (a) no/(b) no; group 2, (a) no/(b) yes; group 3, (a) yes/(b) no; and group 4, (a) yes/(b) yes. The PADRE images were always evaluated in conjunction with the conventional MRI. Furthermore, the reviewers evaluated the slice with the maximum amount of each lateral ventricle. The reading time was unlimited. The images of LBD with or without VH and HS were presented in a blinded and randomized manner for each side of the OR. The reviewers evaluated the PADRE images for abnormal appearances and scored these findings as either positive or negative.

They separated the abnormal OR positive (abnormal OR on at least one side) or negative group (abnormal OR on both sides) for 110 patients with LBD without VH. Moreover, they recognized the appearance of VH in each group during the 5-year follow-up.

### T2WI and FLAIR evaluation

The reviewers, who were blind to the diagnosis, clinical data, and PADRE findings, reviewed the MR studies and categorized the findings (brain atrophy and white matter hyperintensity [WMH]) by consensus. Brain atrophy was defined as deep (enlargement of the ventricles) or peripheral (enlargement of the gyri) and rated on a subjective scale of 0 to 3 (0 = absent, 1 = mild, 2 = moderate, 3 = severe)^15,17^. WMH was graded from 0 to 3 according to the Fazekas scale (0 = none; 1 = single punctate; 2 = multiple punctate; early confluent; 3 = large confluent)^16,18^. For the subjective scale of brain atrophy, “grades 2 or 3” were defined as pathologic brain atrophy. We also divided the patients into two groups based on high or low Fazekas WMH burden (Fazekas grade 0,1 vs. grade 2,3).

### Statistical analyses

We calculated the sensitivity and specificity of the four OR patterns for discrimination between the patients with LBD, with and without VH. This enabled the detection of abnormal OR findings. Furthermore, we calculated the sensitivity and specificity of the abnormal OR findings for each case to diagnose patients with LBD and VH. We considered the strength of interobserver agreement of the abnormal OR findings fair, moderate, good, and excellent for κ values of 0.21–0.40, 0.41–0.60, 0.61–0.80, and ≥ 0.81, respectively. We compared the brain atrophy and Fazekas WMH burden between patients with LBD, with and without VH groups using the × 2 test and Fisher’s exact test, as appropriate.

## Result

Table 1 summarizes the demographic and clinical data of the subjects.

**Table 1:** Demographic and clinical data of the subjects.

		LBD with VH (n = 38)	LBD without VH (n = 110)	HS (n = 18)
LBD	PD	1	92	–
	PDD	9	11	
	DLB	28	7	
Sex, M/F		21/17	36/74	7/11
Age, years (range, mean ± SD)		59–92, 75.9 ± 8.0	46–90, 71.7 ± 9.4	51–86, 68.0 ± 10.1
Disease duration, years (range, mean ± SD)		0.1–20.2, 4.9 ± 4.4	0.1–20.8, 2.9 ± 3.0	–
Medication	Anti-Parkinson’s disease drug	19 (50.0%)	71 (64.5%)	–
	Anti-dementia drug	8 (21.8%)	4 (3.6%)	–
Cognitive impairment		12 (31.6%)	9 (8.2%)	–

*LBD* Lewy body disease, *VH* visual hallucination, *HS* healthy subject(s), *PD* Parkinson's disease, *PDD* Parkinson's disease with dementia, *DLB* dementia with Lewy's bodies, *M* male, *F* female, *SD* standard deviation.

### Nonblinded evaluations

#### Normal appearance of the OR

Coronal PADRE images were acquired covering the entire OR using a 3 T magnetic resonance system. The radiologists evaluated the PADRE for the delineation of the aforementioned three layers at the OR (Fig. 1a). This was based on (external sagittal stratum, internal sagittal stratum, and tapetum) the anatomic appearances of the cadaveric specimens stained with the Bodian's method and the Kluver-Barrera method^11^. The radiologists consensually defined the OR as abnormal in patients with LBD and VH on observing the previously mentioned findings were both present: (a) obscuration of three layers in OR and (b) disappearance of the cranial side of OR.

#### Blinded evaluations of the OR

The sensitivity/specificity of group1 was 29%/30% and 26%/26% for the radiologist 1 and 2, respectively. Those for group 2, 3, and 4 were 1%/95% and 8%/97%, 8%/89% and 9%/90%, and 62%/86% and 57%/86%, respectively. The κ value for interobserver variabilities between the two radiologists was 0.714, indicating good interobserver agreement. The observer study by the two reviewers demonstrated that the sensitivity/specificity of abnormal OR findings was 68%/81% and 66%/80% for each case (LBD with VH vs. LBD without VH) (Table 2).

**Table 2:** Blinded evaluation in radiologist 1.

ORs		LBD with VH	LBD without VH	HS	LBD with VH vs. LBD without VH	
		n = 76	n = 220	n = 36	Sensitivity	Specificity
Group 1	(a) No/(b) no	22	155	32	(29)	(30)
		(29)	(70)	(89)		
Group 2	(a) No/(b) yes	1	10	0	(1)	(95)
		(1)	(5)	(0)		
Group 3	(a) Yes/(b) no	6	24	4	(8)	(89)
		(8)	(11)	(11)		
Group 4	(a) Yes/(b) yes	47	31	0	(62)	(86)
		(62)	(14)	(0)		

Abnormal OR^a^		n = 38	n = 110	n = 18		
Positive		26	21	0	(68)	(81)
		(68)	(19)	(0)		
Negative		12	89	18		
		(32)	(81)	(100)		

Data are raw data; numbers in parentheses are the percentages.

*LBD* Lewy's body disease, *VH* visual hallucination, *HS* healthy subject(s), *OR* optic radiation.

^a^The abnormal OR positive group (the abnormal OR on at least one side) or abnormal OR negative group (the abnormal OR on both bilateral sides).

### Follow-up observation for 5 years

**Figure 2:**
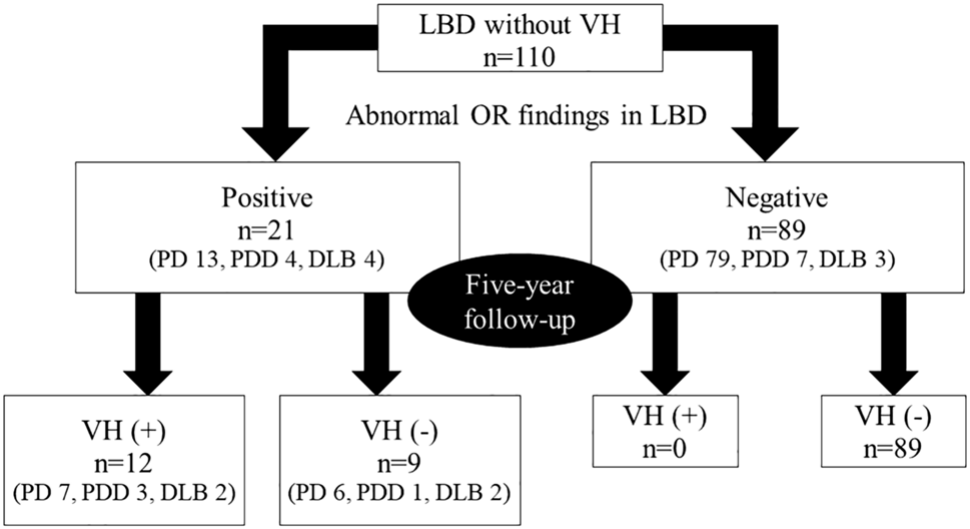
Flow chart of follow-up observation for 5 years (radiologist 1).

VH was recorded in 12 of the 21 (57%) and 11 of the 22 (50%) patients with LBD and abnormal OR findings by the radiologist 1 and 2, respectively. However, no patients without abnormal OR findings experienced VH (Fig. 2, radiologist 1). The new appearance of VH was found in one more case after the follow-up (5.8 years after MRI examination).

### T2WI and FLAIR evaluation of brain atrophy and WMH

There were no significant differences between LBD with VH and LBD without VH groups in pathological brain atrophy (18.4% vs. 9.1%, p = 0.12) and in high Fazekas WMH burden (47.3% vs. 33.6%, p = 0.13). The categories of brain atrophy were as follows: Category 0 (n = 13 vs. 47), 1 (n = 18 vs. 53), 2 (n = 7 vs. 10), 3 (n = 0 vs. 0) in patients with LBD with VH (n = 38) vs. LBD without VH (n = 110). The categories of Fazekas WMH burden were as follows: Category 0 (n = 6 vs. 30), 1 (n = 14 vs. 43), 2 (n = 14 vs. 30), 3 (n = 4 vs. 7) in patients with LBD with VH vs. LBD without VH groups.

## Discussion

We found an association between abnormal OR findings on PADRE and VH in patients with LBD: obscuration of three layer in OR, and disappearance of the cranial side of OR. The observer study with these abnormal findings of the OR demonstrated the sensitivity/specificity were 68%/81% and 66%/80% in radiologist 1 and 2 (LBD with VH vs. LBD without VH). Despite the relatively low sensitivity during five-year follow-up period, the radiologists identified VH in 12 out of the 21 (radiologist 1) and in 11 out of the 22 patients (radiologist 2) with LBD and abnormal OR findings, respectively. However, VH did not appear in patients without abnormal OR findings. Our results suggest that abnormal PADRE appearances might precede the development of VH in patients with LBD. Therefore, structural abnormalities in OR might be associated with VH in patients with LBD. Moreover, OR changes on PADRE might act as a useful marker to identify the presence of VH in patients with LBD and to facilitate the early diagnosis of PDD and DLB.

Some of the PADRE images might offer a new level of contrast that cannot be obtained using any of the previously used phase techniques. According to a previous report, the high-spatial-resolution 3 T PADRE images delineated various fiber tracts of the brainstem as low signal intensity bands^10,11^. Myelination of the nerve fibers was speculated to be one of the factors that determined the low signal intensity on PADRE. Therefore, the PADRE appearances in patients with LBD and VH, obscuration, and the disappearance of the OR might reflect reduced myelination of the OR. This can be attributed to the degeneration of nerve fibers throughout the visual pathways^8^. Lee et al. demonstrated OR abnormality in patients with PD and VH by a quantitative assessment with diffusion tensor imaging data. They measured the fractional anisotropy (FA) and mean diffusivity (MD) values. However, pathological abnormalities in the OR cannot be suspected or diagnosed only by quantitative assessments in clinical practice. This can be attributed to the variation in the measurement of the FA and MD values among individuals. Furthermore, the prevalence of VH increases with the progress of PD^19^. The presence of VH is predictive of poor outcomes, such as those related to cognitive impairment, institutionalization, and higher mortality^5^. Therefore, the PADRE-based assessment, which are clinically available by conventional MRI, might be a simple objective method to evaluate VH in LBD on an individual basis.

Clinicians should consider pathologic changes throughout the visual pathways from the retina to the occipital cortex for understanding the mechanism of obscure OR in patients with LBD. We speculate the degeneration of the OR in both visual afferent neurons (retina, optic nerve, and lateral geniculate nucleus) and efferent neurons (visual cortex) in LBD. Researchers have reported reduced dopamine content and dopaminergic cells in the retina in PD^20^. Moreover, studies using optical coherence tomography have revealed inner retinal layer thinning in PD^21^. Other studies have reported on RNFL thinning in PD^22^. In addition, this retinal change is correlated with the duration and severity of PD^23^ and with VH in non-demented patients with PD^8^. The exact mechanism of inner retinal layer and RNFL thinning is yet to be understood. However, a recent histopathological study has provided evidence for the same. Therefore, the weakening of the retina-optic nerve and lateral geniculate signals because of retinal involvement through PD-related pathology might result in OR degeneration. This might be a direct consequence of the loss of signal synchrony. Several reports have shed light on the role of posterior brain regions, including the visual cortex, in the pathogenesis of VH in LBD. Previous imaging studies in PD patients have reported on hypoactivation of the visual cortex in response to visual stimuli^24^, visual cortex atrophy^25^, and hypometabolism^26^ in the dorsal and ventral visual pathways. Pizzi et al. performed a comparison between DLB and AD-evidenced cortical thinning in the DLB group in the posterior regions^27^. In addition, the correlation analysis between the cortical thickness and neuropsychiatric inventory (NPI) hallucination item scores revealed that a structural alteration in the dorsal visual regions, including the superior parietal gyrus and precuneus, is closely correlated with the occurrence and severity of VH^27^. Therefore, the retrograde degeneration after impairment of the posterior brain regions in LBD, concomitant with the disease progression, might have also affected the OR. Furthermore, in pathological features according to clinical phenotypes, the PD phenotype had brainstem-predominant LBD with scarce cerebral β-amyloid deposition. In contrast, PDD and DLB phenotypes had many cortical Lewy bodies and abundant cerebral amyloid-β deposition. Previous studies have suggested that cerebral β-amyloid accumulation enhances the development of cortical α-synuclein lesions^28,29^, which may influence the neuropathological differences between PD and PDD/DLB. In this study, the patients with LBD without VH group included more patients with PD than with PDD and DLB; the presence of abnormal OR may reflect the cerebral β-amyloid deposition.

According to the non-blinded evaluations by the neuroradiologists, the cranial side of the OR was more frequently absent in patients with VH, compared to the caudal side. Despite the reason for the aforementioned finding being unclear, we speculated on a possible mechanism. Previous neuroimaging^30^, neuropathological^31^ and electrophysiological studies^32^ have revealed that structural abnormalities in the cuneus and the higher visual areas related to VH in the DLB group. A previous study reported on both the dorsal and ventral attention networks being affected in patients with DLB, compared to controls and those with AD^27^. However, the correlation analysis between the cortical thickness and NPI hallucination item scores demonstrated a greater involvement of the dorsal attention regions, including the superior parietal gyrus and precuneus, in the occurrence and severity of VH in DLB^27^. The superior parietal region is implicated in visuospatial working memory^25^, retrieval and representations^30^, and processing of visuospatial tasks^33^. Therefore, deficits in visual attention and in dorsal attention network superior parietal gyrus and precuneus have been considered important factors in the causal models of VH using a voxel-based morphometry analysis^34^. Hence, our abnormal findings in the cranial side of the OR on PADRE might reflect the secondary neurodegenerative changes because of structural abnormalities in the superior parietal gyrus and precuneus.

The present study showed no significant differences between LBD with VH and LBD without VH groups in brain atrophy and WMH. These results were consistent with previous reports that have examined whether brain atrophy or WMH are associated with VH in patients with PD^35,36^. Although it should be noted that VH is likely of multifactorial origin, our study may support a growing body of literature linking VH to the pathology of the visual system.

Our study had several limitations. First, we adopted a retrospective design. Many patients with LBD received medication before undergoing MR imaging, and the type of medicine, dose, and history of medications are not standardized. Furthermore, cognitive assessments were not administered to all patients with LBD, and the tests were not standardized. Therefore, it was not possible to exclude the influence of medications or cognitive impairment on the abnormal findings in OR. In addition, since we couldn’t obtain detailed information about VH, including contents, severity, and frequency, we didn’t evaluate the pattern of VH related to the abnormal OR findings on PADRE. Second, we did not perform ophthalmologic examinations. Moreover, the unavailability of 3-D MRI data in several patients resulted in our failure to evaluate the brain volume in the visual areas and the volume of WMH. Finally, this study could not assess whether the changes in retinal and visual cortical regions were anterograde or retrograde to abnormal OR in patients with LBD. Therefore, future research should be performed to investigate the longitudinal associations assessing the degeneration of nerve fibers throughout the visual pathways from the retina to the cerebral cortex considering the clinical or demographic heterogeneity of the patients with LBD.

In conclusion, we found new abnormal OR findings in patients with LBD and VH on PADRE. The findings are likely to be useful in distinguishing between patients without VH and HS. Moreover, abnormal OR on PADRE in patients with LBD may precede the appearance of VH. Future research should be performed to investigate whether abnormal OR on PADRE can be used as an imaging biomarker for predictor of the appearance of VH in patients with LBD.

## Data Availability

The datasets generated and/or analyzed during the current study are not publicly available due to the anonymity of the patients but are available from the corresponding author on reasonable request.
